# Case report: Diagnosis, management and evolution of a bulky and invasive cardiac mass complicated by complete atrioventricular block

**DOI:** 10.3389/fcvm.2023.1135233

**Published:** 2023-03-14

**Authors:** Yohann Bohbot, Jérôme Garot, Isabelle Danjon, Dominique Thébert, Louis Nahory, Philippe Gros, Fiorella Salerno, Philippe Garot

**Affiliations:** ^1^ Institut Cardiovasculaire Paris Sud, Hôpital Privé Jacques CARTIER, Ramsay Santé, Massy, France; ^2^ Intensive Care Unit Department, Hôpital Privé Jacques CARTIER, Ramsay Santé, Massy, France; ^3^ Department of Pathology, Praxea Diagnostics, Massy, France

**Keywords:** lymphoma, atrioventicular block, cardiac magnet resonance imaging (CMR), biopsy, chemotherapy

## Abstract

**Introduction:**

Cardiac lymphoma is a rare but serious disease that is usually located in the right heart. The symptoms (dyspnea, respiratory distress, fatigue, syncope…) are not specific and depend on the mass location. Cardiac magnetic resonance has a crucial role in the diagnostic strategy but biopsy is mandatory to confirm the diagnosis.

**Case presentation:**

We report the case of a 63-yeart old man who presented with severe dyspnea and complete atrioventricular block (AVB). A bulky and invasive mass was found in the left atrium extending to the right atrium through the interatrial septum. A cardiac lymphoma was suspected by cardiac magnetic resonance (CMR) imaging and confirmed by transvenous biopsy. The patient was treated with urgent chemotherapy (R-CHOP) and pacemaker implantation. After 4 cycles of R-CHOP the patient was in complete remission with total disappearance of the mass and return of a spontaneous sinus rhythm.

**Conclusion:**

lymphoma is a therapeutic emergency as appropriate treatment can lead to complete remission even when the mass is extensive and invasive. Complete AVB is a potentially reversible complication of cardiac lymphoma, and the decision to implant a pacemaker must be carefully weighed.

## Introduction

Multimodality imaging, and in particular cardiac magnetic resonance (CMR), plays a key role in the diagnostic strategy of cardiac masses (1, 2). However, when a malignancy is suspected, a cardiac biopsy is necessary to distinguish sarcoma from lymphoma, as the treatments are diametrically opposed (1). Lymphomas are most often located in the right heart and rarely involves the left side, but few cases of left heart localization have been reported (3, 4). To our knowledge, we report the first case of a large left-sided cardiac lymphoma complicated by complete atrioventricular block (AVB) that was diagnosed by transvenous imaging-guided biopsy through its extension across the interatrial septum. Our case also illustrates that cardiac lymphoma is a therapeutic emergency, as a rapid intravenous bolus of corticosteroids and chemotherapy can result in rapid regression of the mass even when the mass is extensive and invasive, and that complete AVB is a potentially reversible complication of cardiac lymphoma.

## Case presentation

**Figure 1:**
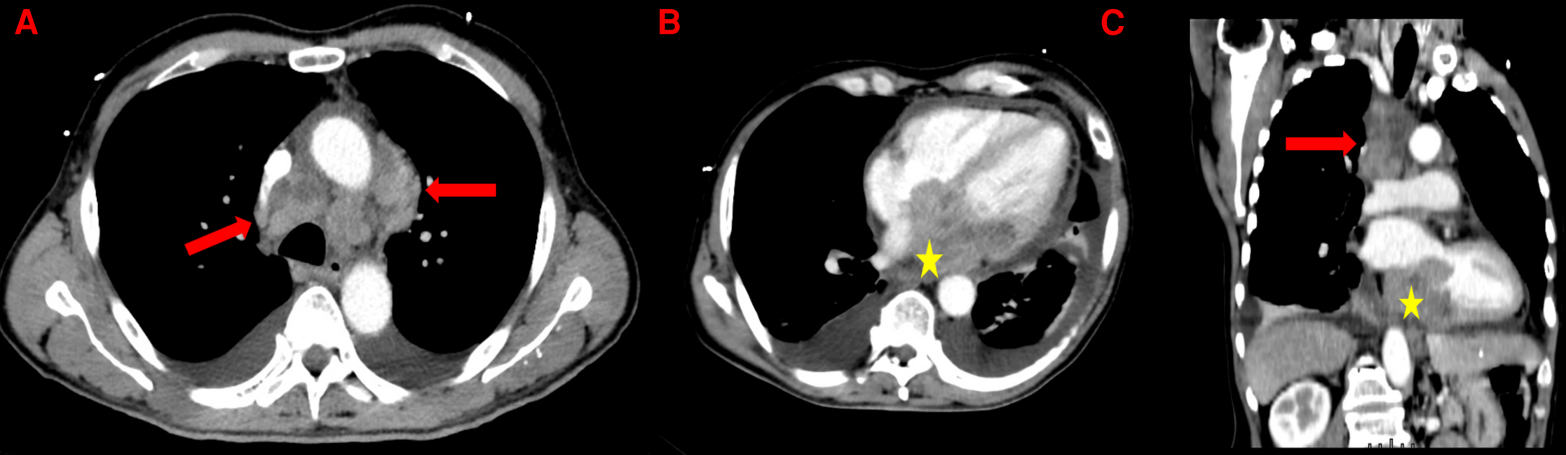
Thoracic CT-scan showing mediastinal lymphadenopathies (red arrows) and the left atrial mass (yellow stars) in axial view (**A** and **B**) and in coronal view (**C**). Presence of a bilateral pleural effusion and a thickened pericardium (**B**).

**Figure 2:**
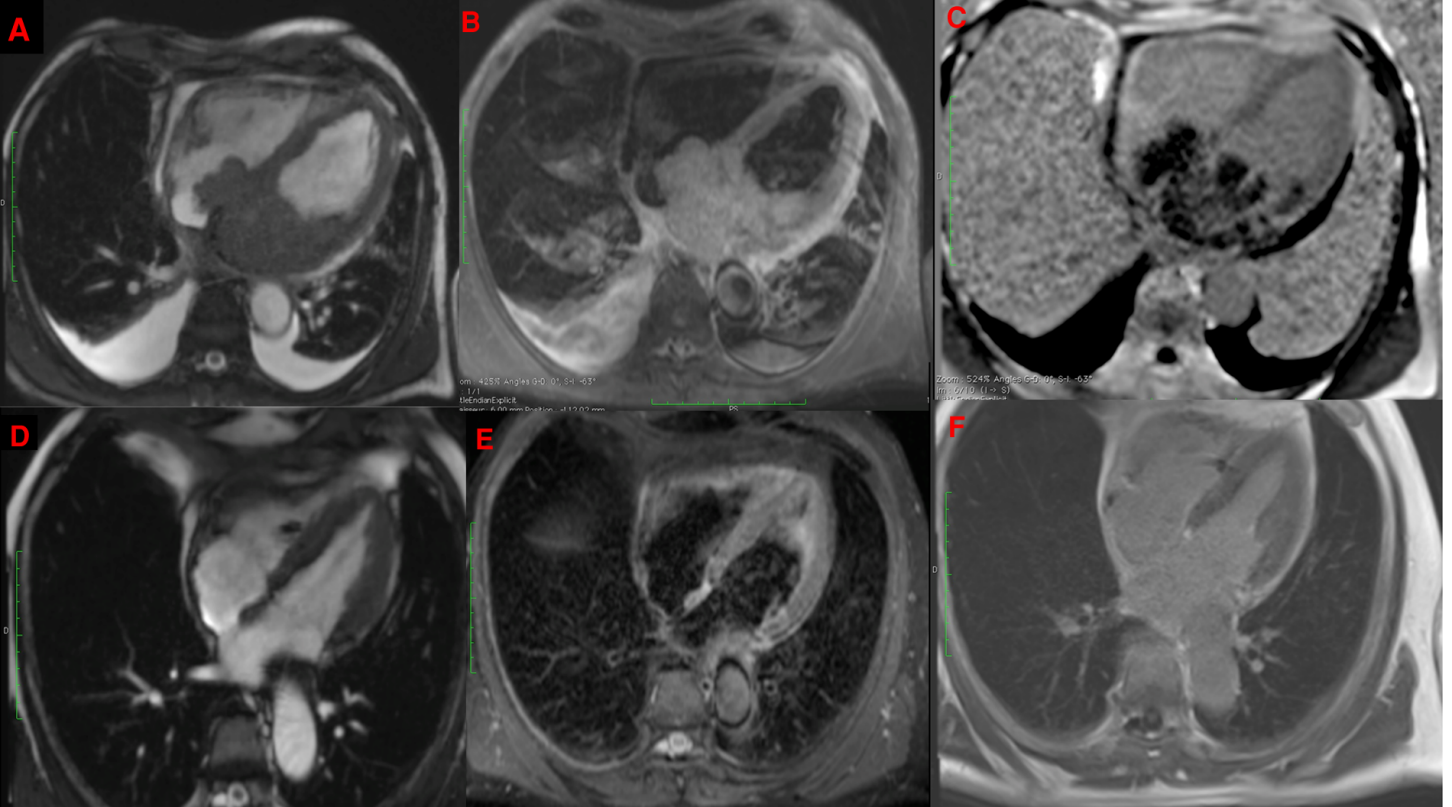
CMR showing a bulky mass extending throughout the left atrium, infiltrating the interatrial septum and right atrium, on cine imaging sequence (**A**), slightly hyperintense on fat-saturated T2-weighted imaging (**B**) with heterogeneous enhancement on late gadolinium enhancement sequences (**C**) with complete disappearance after 6 cycles of R-CHOP in the same sequences (**D–F**).

**Figure 3:**
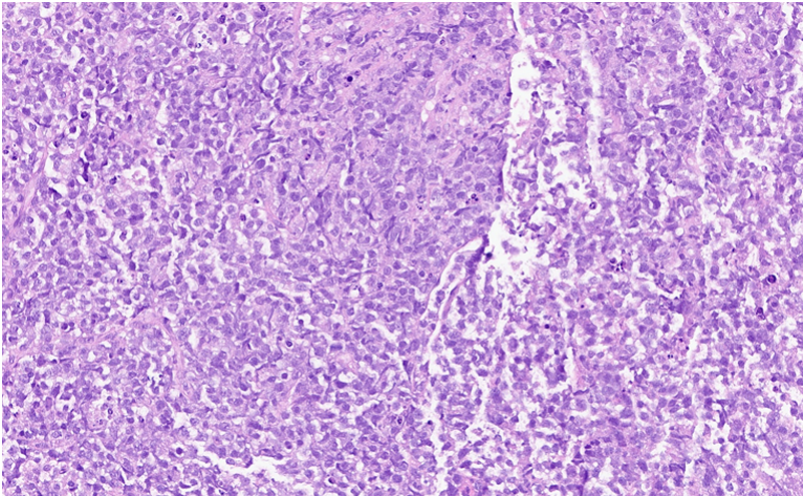
Hematoxylin and eosin stain: large lymphocytes cells with moderately abundant cytoplasm and oval nuclei containing several nucleoli corresponding to centroblasts.

In March 2022, a 63-year-old man without notable past medical history presented with rapidly progressive exertional dyspnea associated with night sweats. The electrocardiogram revealed complete atrioventricular block (AVB) with a heart rate of 40 beats per minutes and narrow QRS complexes. Lab tests showed anemia with hemoglobin at 9 g/dl, normal renal function and a biological inflammatory syndrome with CRP at 181 mg/L (normal upper limit: 4 mg/L) and the patient was transferred to the intensive care unit with Isoprenaline infusion. The initial workup included a bedside transthoracic echocardiography, which revealed a heterogeneous left atrial mass, measured at 50 × 60 mm, infiltrating the inter-atrial septum with an extension into the right atrium measured at 43 × 26 mm. This mass seemed to be implanted in the lateral wall of the left atrium and prolapsed into the mitral valve causing moderate mitral regurgitation and significant mitral stenosis with a mean gradient ranging from 7 to 14 mmHg. Then, a thoracic, abdominal and pelvic CT scan was performed and showed the cardiac mass associated with a mild pericardial effusion, hilar and mediastinal lymphadenopathy and bilateral moderate pleural effusion (Figure 1). The workup was completed by a cardiac magnetic resonance imaging (CMR) study to better characterize the mass. CMR confirmed the presence of a bulky mass with a “cauliflower” appearance, extending throughout the entire left atrium, infiltrating the interatrial septum and the right atrium as well as the basal inferior and inferolateral walls of the left ventricle (Figure 2A). This mass appeared isointense on black blood spin echo T1-weighted imaging, slightly hyperintense on fat saturated T2-weighted imaging with a heterogeneous enhancement on late gadolinium enhancement (LGE) sequences (Figures 2B,C), suggestive of a malignant tumor. In order to distinguish between cardiac lymphoma or sarcoma, for which initial treatments are diametrically opposed (i.e., urgent chemotherapy vs. surgical resection) (1), a myocardial biopsy guided by transesophageal echocardiography was performed. Four endomyocardial fragments were collected from the right atrial side of the interatrial septum *via* a femoral venous access. Given the persistence of complete AVB at day 7, a VVI single lead transvenous pacemaker was implanted. Prompt pathological analysis revealed the presence of a diffuse large B-cell lymphoma classified as germinal center B cell type (CD10+, BCL6+, MUM1+), with a double expression of MYC/BCL2 (Figure 3). Urgent treatment was initiated as soon as the pathology results were obtained, 10 days after the biopsy, with corticosteroids (1 mg/kg) associated with chemotherapy including rituximab, cyclophosphamide, doxorubicin, vincristine, and prednisone (R-CHOP). Six cycles of R-CHOP were completed within 6 months, allowing a clear improvement of the dyspnea and of the overall condition. CT-scan after the fourth cycle of R-CHOP showed complete remission of the lymphoma (2). After setting the MR conditional pacemaker to DOO mode, follow-up CMR revealed complete disappearance of the cardiac mass (Figures 2D–F). Because the patient was in spontaneous sinus rhythm at the last visit and the stimulation rate dropped from 100% to 26% after the third R-CHOP cycle, an evaluation by the cardiac electrophysiologist was scheduled 3 months later to assess the percentage of RV pacing for possible pacemaker removal. Written informed consent was obtained from the patient for the publication of any potentially identifiable images or data included in this article.

## Discussion

Cardiac lymphoma is a rare disease accounting for only 1.3% of all cardiac tumors and 0.5% of lymphomas (1, 3). There is a male predominance and the mean age at diagnosis is 63 years as in the current case (3). The tumor is most often located in the right heart (atrium or ventricle) and rarely involves the left side, but few cases of left heart localization have been reported (3, 4). The symptoms are not specific and depend on the location of the mass. They may include dyspnea with possible respiratory distress, syncope related to cardiac rhythm or conduction disorders, superior vena cava syndrome as well as altered overall condition and fatigue (3). In our patient's case, the dyspnea was most likely related to the obstructive nature of the tumor through the mitral valve resulting in mitral stenosis and to the complete AVB due to conduction pathway infiltration.

Cardiac masses include benign tumors, primary and secondary malignancies, and other diagnoses (e.g., thrombus, pericardial cyst, and Lambl's excrescences). Multimodality imaging, and in particular CMR, plays a key role in the diagnostic strategy (1) Beyond the localization and mass aspect, T1 and T2 weighted imaging and LGE sequences are crucial to distinguish between benign from malignant tumors (1, 5). Lymphoma generally appears isointense on T1- and T2-weighted imaging (or mildly T2-hyperintense due to diffuse edema) and may or may not show enhancement on LGE sequences. Sarcomas also appear isointense on T1-weighted imaging but show hyperintensity on T2-weighted imaging and heterogeneous enhancement on LGE sequence (1, 5). Because of the slight T2 hyperintensity in our patient, both diagnoses were possible. Nevertheless, the distinction between these two entities is mandatory, because the treatments and outcomes are totally different. Complete surgical resection, that is often difficult and damaging to the cardiac structures is the best option for sarcomas, whereas corticosteroid therapy and chemotherapy should be initiated promptly for cardiac lymphoma (1). Corticosteroids can be started promptly without waiting for the biopsy results if there is a strong clinical suspicion of lymphoma in order to improve the patient's symptoms (6). Surgery is not indicated in the management of cardiac lymphoma except for pathological documentation when transvenous biopsy is not feasible (1). Indeed, histological documentation is required and essential to establish the correct diagnosis and to start the appropriate treatment without delay. Fortunately for our patient, the mass had invaded the right atrium through the interatrial septum and an imaging-guided transfemoral biopsy was possible, which is rarely the case for left heart masses.

Primary cardiac lymphoma in immunocompetent patients −as in this case− are infrequent, accounting for only 1.3% of primary cardiac tumors (1, 7). The vast majority of cardiac lymphomas are aggressive B-cell lymphomas carrying a poor prognosis, with more than 60% of patients dying within 2 months of diagnosis (1, 8). Predictors of unfavorable outcome include immune status, presence of extracardiac disease, left ventricular involvement, and arrhythmia (3). However, the response rates to chemotherapy ranged between 79% and 87% and unlike cardiac sarcomas, numerous other cases of complete remission have been reported in cardiac lymphomas (9). Most cases of complete remission of cardiac lymphoma involve the right heart, but some cases involving the left side, diagnosed and treated rapidly as in the present one, have also been reported (4, 9).

Complete AV block is a known complication of cardiac tumors, and few cases have been reported in cardiac lymphomas (10, 11). In most of the reported cases, these patients benefited from pacemaker implantation but the conduction disorders improved under chemotherapy, raising the question of the need for permanent pacemaker implantation in this case (10, 11). If the patient is clinically stable, it may be reasonable to delay the pacemaker implantation until evaluation of the clinical response to chemotherapy or to use a temporary pacemaker in the meantime (10). Subramanyam et al. proposed an algorithm for the management of cardiac conduction abnormality in patients with cardiac lymphoma (11). They suggest, in the absence of hemodynamic instability, to wait until performing CMR to assess the extent of cardiac involvement by the tumor before placing the temporary pacemaker. Then, two decisions should be taken: the duration of the temporary pacing before the implantation of a definitive pacemaker and the type of pacemaker to be implanted (i.e., transvenous or leadless) (11). Transition to a permanent pacemaker should be considered in cases of persistent complete AVB with extensive lesions impacting cardiac conduction that are not expected to resolve in a few weeks, as in our patient's case (11) There are no recommendations on the type of pacemaker that should be implanted and, if dual chamber pacing is not required, a leadless pacemaker could be considered in these patients who are at high risk of infection (11). However, no study has yet compared transvenous and leadless pacemakers in cancer patients in terms of complication rates. Thus, the management of cardiac conduction disorders associated with lymphoma is complex and requires close coordination between cardiology and oncology teams. In our patient's case, the pacing rate dropped from 100% to 26% after the third cycle of R-CHOP, and a reevaluation by the cardiac electrophysiologist was planned to discuss possible removal of the device.

Despite the complete remission, the patient will be followed closely by cardiac and thoracic imaging with rigorous hematological monitoring because recurrence risk (often extracardiac) estimated at 55% (9).

## Conclusions

**Figure 4:**
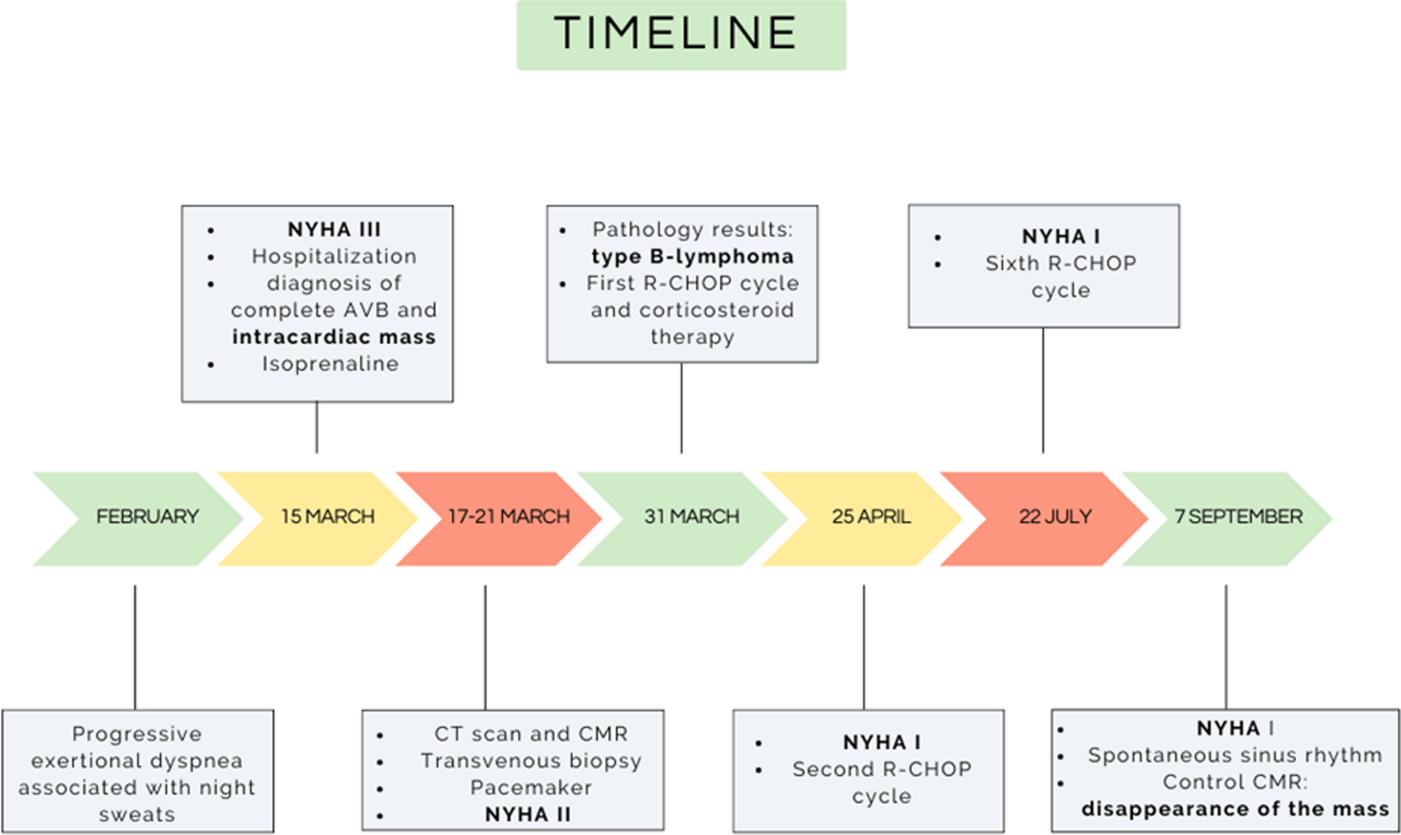
Timeline of the case. AVB, atrioventricular block; CMR, cardiac magnetic resonance; CT, computed tomography; NYHA, New York Heart Association.

A prompt cardiac biopsy, ideally percutaneous and guided by imaging should be performed if a malignant cardiac tumor is suspected in order to select the appropriate treatment. Cardiac lymphoma should be considered as a therapeutic emergency, since prompt intravenous bolus of corticosteroids and chemotherapy can provide rapid regression of the mass and ultimately complete remission even when the mass is extensive and invasive. Complete AVB is a potentially reversible complication of cardiac lymphoma, and the decision to implant a pacemaker must be carefully weighed (Figure 4).

## Data Availability

The original contributions presented in the study are included in the article/Supplementary Material, further inquiries can be directed to the corresponding author.
